# Bisphenol A Exposure during Adulthood Causes Augmentation of Follicular Atresia and Luteal Regression by Decreasing 17β-Estradiol Synthesis via Downregulation of Aromatase in Rat Ovary

**DOI:** 10.1289/ehp.1205823

**Published:** 2013-03-19

**Authors:** Seung Gee Lee, Ji Young Kim, Jin-Yong Chung, Yoon-Jae Kim, Ji-Eun Park, Seunghoon Oh, Yong-Dal Yoon, Ki Soo Yoo, Young Hyun Yoo, Jong-Min Kim

**Affiliations:** 1 Department of Anatomy and Cell Biology, College of Medicine, Dong-A University, Busan, Korea; 2 Mitochondria Hub Regulation Center, and; 3 Medical Science Research Center, Dong-A University, Busan, Korea; 4 Department of Physiology, College of Medicine, Dankook University, Cheonan, Korea; 5 Department of Life Science, College of Natural Sciences, Hanyang University, Seoul, Korea

**Keywords:** 17β-estradiol, aromatase, bisphenol A, follicular atresia, luteal regression, ovary, steroidogenic acute regulatory protein

## Abstract

Background: Bisphenol A (BPA) has been detected in human body fluids, such as serum and ovarian follicular fluids. Several reports indicated that BPA exposure is associated with the occurrence of several female reproductive diseases resulting from the disruption of steroid hormone biosynthesis in the adult ovary.

Objective: We hypothesized that long-term exposure to low concentrations of BPA disrupts 17β-estradiol (E2) production in granulosa cells via an alteration of steroidogenic proteins in ovarian cells.

Methods: Adult female rats received BPA for 90 days by daily gavage at doses of 0, 0.001, or 0.1 mg/kg body weight. We determined serum levels of E2, testosterone (T), follicle-stimulating hormone (FSH), and luteinizing hormone (LH). We also analyzed the expressions of steroidogenic acute regulatory protein (StAR), P450 side-chain cleavage (P450scc), 3β-hydroxysteroid dehydrogenase isomerase (3β-HSD), and aromatase cytochrome P450 (P450arom) in the ovary.

Results: Exposure to BPA significantly decreased E2 serum concentration, which was accompanied by augmented follicular atresia and luteal regression via increase of caspase-3–associated apoptosis in ovarian cells. After BPA exposure, P450arom and StAR protein levels were significantly decreased in granulosa cells and theca-interstitial (T-I) cells, respectively. However, P450scc and 3β-HSD protein levels remained unchanged. The increase in LH levels appeared to be associated with the decreased synthesis of T in T-I cells after BPA exposure via homeostatic positive feedback regulation.

Conclusions: BPA exposure during adulthood can disturb the maintenance of normal ovarian functions by reducing E2. The steroidogenic proteins StAR and P450arom appear to be targeted by BPA.

Bisphenol A [BPA; 2,2-bis-(4-hydroxyphenyl)propane] is a plasticizer that is widely used to produce polycarbonate plastic, epoxy resin, and unsaturated polystyrene. BPA can leach from the linings of food cans, polycarbonate baby bottles and other beverage containers, dental sealants and composites, polyvinyl chloride plastics, and recycled thermal paper, resulting in human exposure to BPA (Vandenberg et al. 2007). In fact, BPA has been detected in human serum (Takeuchi and Tsutsumi 2002), urine (Calafat et al. 2008), breast milk (Ye et al. 2006), and ovarian follicular fluids (Ikezuki et al. 2002). The increased incidence of BPA exposure in humans is suspected to be associated with the occurrence of various reproductive diseases and health outcomes including male sexual dysfunction (Li et al. 2010), recurrent miscarriage (Sugiura-Ogasawara et al. 2005), premature delivery (Cantonwine et al. 2010), and polycystic ovary syndrome (PCOS) (Kandaraki et al. 2011).

In animal experiments, BPA exposure has been shown to have adverse effects on the reproductive system (Hunt et al. 2009). In female reproduction, neonatal or perinatal exposure to BPA has been reported to cause significant histological changes in the reproductive tract (Newbold et al. 2007, 2009), alteration of estrous cyclicity (Rubin et al. 2001), decreased reproductive capacity (Cabaton et al. 2011), and changes in hormonal levels (Fernández et al. 2009; Rubin et al. 2001) later in adult life. In the ovary, disruption of follicular development (Adewale et al. 2009), reduction of the pool of primordial follicles (Rodríguez et al. 2010), and the occurrence of PCOS-like structures (Fernández et al. 2010) have been observed after neonatal exposure to BPA. However, the effect of BPA exposure on the alteration of ovarian steroidogenesis in adult animals has not been elucidated. BPA treatment has been reported to alter steroid hormone production in granulosa cells (Grasselli et al. 2010; Mlynarcíková et al. 2005; Zhou et al. 2008). Furthermore, the effect of BPA on steroidogenesis has been demonstrated in a mouse follicle culture system (Peretz et al. 2011). Nevertheless, the precise cellular and biochemical mechanism(s) by which BPA affects ovarian steroidogenesis have not yet been identified in animals that are chronically exposed to BPA during adulthood.

We hypothesized that the adult ovary is susceptible to BPA *in vivo* and that long-term exposure to low concentrations of BPA disrupts 17β-estradiol (E2) production by granulosa cells via the alteration of steroidogenic proteins in ovarian cells, such as steroidogenic acute regulatory protein (StAR), P450 side-chain cleavage (P450scc), 3β-hydroxysteroid dehydrogenase isomerase (3β-HSD), cytochrome P450 17A1 (CYP17A1), and aromatase cytochrome P450 (P450arom). To test this hypothesis, we examined the expression levels of these proteins in relation to serum E2 levels and evaluated cellular and histological alterations in the ovary.

## Materials and Methods

*Materials*. Monoclonal anti-actin (mouse IgG2a isotype), anti-β-tubulin, BPA, Bouin’s solution, corn oil, dimethyl sulfoxide (DMSO), hematoxylin, HEPES, medium 199, trypan blue, and Tween-20 were purchased from Sigma Chemical Co. (St. Louis, MO, USA). We purchased anti-aromatase from Acris Antibodies (San Diego, CA, USA); anti-calbindin-D9k from Swant Swiss Antibodies (Bellinzona, Switzerland); antibody specific for cleaved (active form) caspase-3 antibody from Cell Signaling (Beverly, MA, USA); anti-FSH (follicle-stimulating hormone) antibody from AbD Serotec (Kidlington, UK); anti-3β-HSD, anti-CYP17A1, proliferating cell nuclear antigen (PCNA), and rabbit IgG antibodies from Santa Cruz Biotech (Delaware, CA, USA); anti-P450scc antibody from Chemicon (Temecula, CA, USA); and anti-StAR and anti-LH (luteinizing hormone) antibodies from Abcam (Cambridge, UK).

*Animals and BPA exposure*. Adult female Sprague-Dawley rats [8 weeks of age, 200–250 g body weight (BW)] were purchased from SamTako Bio-Korea (Osan, Korea). The rats were housed in a climate-controlled (21 ± 2°C) animal room at a constant 12-hr light/dark cycle, with unlimited access to rat chow. All procedures were performed in accordance with protocols approved by the Dong-A University Animal Care and Use Committee. The animals were treated humanely and with regard for alleviation of suffering. The rats received BPA daily for 90 days by gavage at doses of 0.001 (low dose) or 0.1 (high dose) mg/kg BW (*n* = 30 rats/dose). The estrogenic control group (*n* = 30) received estradiol benzoate (EB; 0.001 mg/kg BW) instead of BPA. Control animals (*n* = 30) received the same weight-based volume of vehicle (0.5% DMSO in corn oil). The lowest observed adverse effect level (LOAEL) for BPA established by the U.S. Environmental Protection Agency (EPA) is 50 mg/kg BW/day, and the U.S. EPA reference dose (and the U.S. Food and Drug Administration acceptable daily intake) is 50 μg/kg BW/day (U.S. EPA 1993).

After 90 days of daily gavage, a portion of the rats (*n* = 18/dose) were sacrificed by carbon dioxide asphyxiation on the day of the normal estrus phase [routinely identifiable by the presence of large numbers (≥ 50%) of needle-like, cornified (or keratinized) cells], and the right ovaries and uterine horns were removed and fixed in Bouin’s fixative for histological examination. The left ovaries were placed in cold phosphate-buffered saline (PBS) for collection of granulosa cells, and the left uterine horns were snap frozen for further biochemical analysis. The remaining rats (*n* = 12/dose) were continuously examined for estrous cycle staging.

*Hormone assays*. E2 and T serum concentrations were determined in duplicate samples using E2 and T enzyme-linked immunosorbent assay (ELISA) kits (IBL, Hamburg, Germany) according to the manufacturer’s instructions. The sensitivity of the E2 assay was 9.71 pg/mL, and the intraassay and interassay coefficients of variation (CVs) were 2.7% and 7.2%, respectively. The sensitivity of the T assay was 0.08 ng/mL, and the intraassay and interassay CVs were 3.3% and 6.7%, respectively. We determined serum concentrations of FSH and LH for duplicate samples using FSH (ELIZEN Rat FSH; ZenTech, Angleur, Belgium) and LH (LH DETECT®; INRA, Nouzilly, France) ELISA kits. The sensitivity of the FSH assay was 0.2 ng/mL, and the intraassay and interassay CVs were 4.7% and 8.4%, respectively. The sensitivity of the LH assay was 0.01 ng/mL, and the intraassay and interassay CVs were 4.2% and 8.1%, respectively.

*Estrous cycle staging*. Beginning the day after BPA treatment was completed (day 1), vaginal smears from each animal were collected between 0800 hours and 1000 hours by lavage with 0.9% saline. The fluid was spotted thinly on a microscope slide, and the dried slides were stained with 0.1% trypan blue in deionized water. The estrous cycle stage was determined by microscopic examination, as described by Westwood (2008). We examined vaginal cytology for a total of 30 days: days 1–15 and days 31–45. No vaginal smears were obtained on days 15–30 to avoid mechanical stress on the vaginas of the rats. We determined the proportion of days that rats were in estrus by dividing the total number of estrus days by the number of days the estrous cycle was examined (30 days).

*Granulosa cell isolation and collection of residual ovaries*. Granulosa cells were collected by follicular puncture as described previously (Rao et al. 1991). Briefly, granulosa cells from ovaries were harvested in ice-cold M199 medium supplemented with HEPES (25 mM, pH 7.4) by follicle puncture with a 27-gauge hypodermic needle and centrifuged at 900 × *g* for 5 min, the supernatant was discarded, and the pellet was immediately frozen on dry ice and stored at –80°C. The residual ovaries [retaining theca-interstitial cells (T-I)] were thoroughly washed with M199 to release undissociated granulosa cells, transferred into clean tubes, frozen on dry ice, and stored at –80°C.

*Immunohistochemistry and histochemical staining for collagen fibers*. For immunohistochemical staining of ovary and uterus, tissue sections were deparaffinized and hydrated, treated in 3% hydrogen peroxide for 5 min, and rinsed with PBS for 15 min. Sections were incubated with the primary and secondary antibodies and labeled using the Vectastain ABC kit (Vector Laboratories, Burlingame, CA, USA) according to the manufacturer’s instructions. The nuclei were counterstained with hematoxylin. For negative controls, rabbit IgG (1 mg/mL) was used instead of the primary antibodies. In the ovarian sections, the number of atretic follicles and regressing corpus luteum that retained at least one caspase-3–positive cell was counted and divided by the total number of follicles and corpus luteum, respectively, in order to calculate the incidence (percentage) of follicular atresia and luteal regression. Histochemical staining for collagen fibers in uterine tissues was performed using the ACCUSTAIN Trichrome Stains (Gomory method) kit (Sigma) according to the manufacturer’s instructions.

*Western blot analysis*. We performed Western blot analysis as described previously (Chung et al. 2011). Briefly, granulosa cells were lysed to obtain protein samples. Then proteins (~ 30 μg) were resolved by sodium dodecyl sulfate polyacrylamide gel electrophoresis (SDS-PAGE), transferred onto a nitrocellulose membrane, probed with a primary antibody, and labeled with horseradish peroxidase-labeled secondary antibodies. Signals were detected with an enhanced chemiluminescence detection kit (Amersham Pharmacia Biotech, Piscataway, NJ, USA).

*Statistical analysis*. Data are expressed as the mean ± SD of three or four separate experiments. When appropriate, data were analyzed using analysis of variance followed by Duncan’s post hoc test. Values were considered significantly different at *p* < 0.05.

## Results

**Figure 1:**
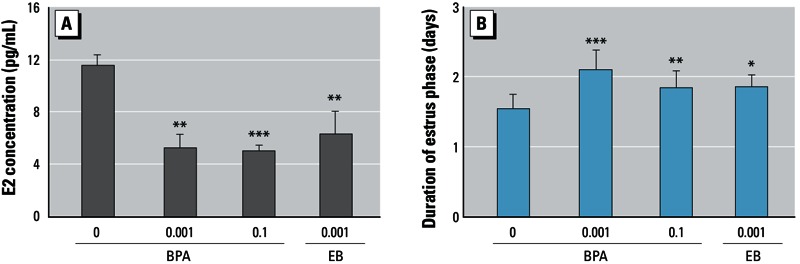
Effect of BPA on E2 serum concentration (*A*) and duration of the estrus phase (*B*). Adult female rats were administered BPA (0, 0.001, or 0.1 mg/kg BWU/day) or EB (0.001 mg/kg BW/day) for 90 days by gavage. E2 levels were measured by ELISA (*A*), and staging of the estrous cycle was determined by a vaginal smear (*B*). Values represent the mean ± SD (*n* = 12). *p < 0.05, **p < 0.01, and ***p < 0.001, compared with control (0 mg/kg BW).

*Effect of BPA on E2 serum concentrations and uterine alterations during estrus.* To determine whether BPA exposure induces alterations of the female reproductive system, we treated adult female Sprague-Dawley rats with BPA at either 0.001 mg/kg BW (low dose) or 0.1 mg/kg BW (high dose) for 90 days by gavage. Both doses of BPA significantly decreased serum E2 concentrations compared with controls (Figure 1A). In contrast, the duration of the estrus phase was extended by BPA exposure (Figure 1B). Exposure to EB resulted in the same alteration patterns as observed with BPA (Figure 1A,B). All of the animals tested for the 45-day period continued to cycle, and most BPA- or EB-treated rats showed an extended estrus phase of 2–7 days. However, no animals entered a persistent estrus phase.

*Reduced E2 levels after BPA were confirmed by alterations in uterine cell proliferation and integrity*. To confirm whether the reduced E2 levels after BPA treatment resulted in uterine tissue alterations, we examined estrogen-reactive proteins related to cell proliferation (PCNA) and tissue integrity (calbindin-D9k and collagens) in the uterus. PCNA proteins were predominantly localized in the nuclei of luminal endometrial cells [see Supplemental Material, Figure S1A, a–d (http://dx.doi.org/10.1289/ehp.1205823)], and the number of PCNA-positive cells was much higher in the control group (see Supplemental Material, Figure S1A, a) than in either the BPA- or EB-treated group (see Supplemental Material, Figure S1A, b–d). The immunoreactivity for calbindin-D9k, an estrogen-responsive protein (Krisinger et al. 1992), in myometrial tissues was much more intense in the control group (see Supplemental Material, Figure S1A, e) than in either the BPA or EB-treated group (see Supplemental Material, Figure S1A, f–h). Downregulation of PCNA and calbindin-D9k protein levels after BPA exposure were confirmed by Western blot analysis (see Supplemental Material, Figure S1B,C). Furthermore, trichrome staining showed that BPA exposure reduced the amount of collagen fibers in the myometrium and endometrium of uterine tissues (see Supplemental Material, Figure S2).

**Figure 2:**
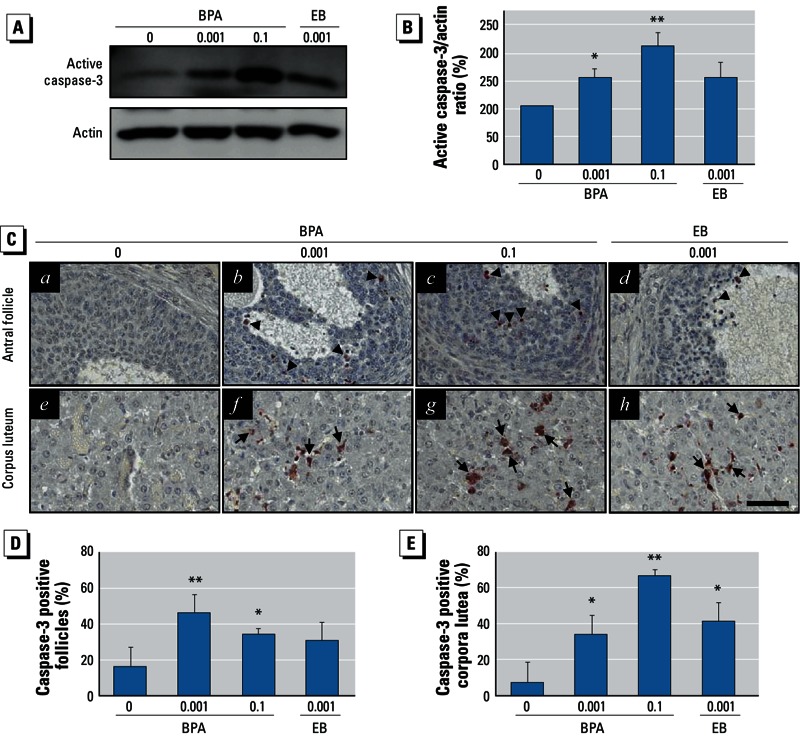
Effect of BPA on caspase-3–associated apoptotic cell death in ovarian cells and follicular atresia augmentation and luteal regression. Adult female rats were administered BPA (0, 0.001, or 0.1 mg/kg BWU/day) or EB (0.001 mg/kg BW/day) for 90 days by gavage. (*A*) Caspase-3-associated apoptotic cell death in the ovaries evaluated by Western blot analysis using an active form-specific caspase-3 antibody. (*B*) Densitometric quantification of activated caspase-3 protein levels in total ovarian protein extracts. For (*A*) and (*B*), data represent the mean ± SD of at least three independent experiments. (*C*) Immunolocalization of active caspase-3 in ovaries. Arrowheads indicate active caspase-3-positive granulosa cells, and arrows point to luteal cells. Original magnification: 200×; bar = 60 μm. Changes in the proportion of atretic follicles (*D*) and the regressing corpus luteum (*E*) in the ovary. For *B*, *D*, and *E*, data represent the mean ± SD of at least three independent experiments. *p < 0.05, and **p < 0.01 compared with control (0 mg/kg BW).

*Increased ovarian cell apoptosis after BPA exposure correlated with augmentation of follicular atresia and luteal regression*. To examine whether reduced E2 levels are associated with increased degenerative processes (i.e., ovarian follicular atresia and luteal regression) in the ovary, we investigated caspase-3–dependent apoptotic cell death in the follicles and corpus luteum. Western blot analysis for caspase-3 in whole ovarian tissues showed that caspase-3 activation was significantly increased by BPA exposure (Figure 2A) in a dose-dependent manner (Figure 2B). Similarly, we observed caspase-3 immunoreactivity more frequently in the granulosa cells of the degenerating (atretic) follicles (Figure 2C, b–c) and in the luteal cells of the corpus luteum (Figure 2C, f–g) in ovaries of the BPA-treated groups compared with controls (Figure 2C, a–e). A significantly higher number of follicles and corpus luteum retained caspase-3–positive cells in ovaries from BPA-treated animals compared with those from controls [Figure 2D,E; see also Supplemental Material, Figure S3 (http://dx.doi.org/10.1289/ehp.1205823)].

**Figure 3:**
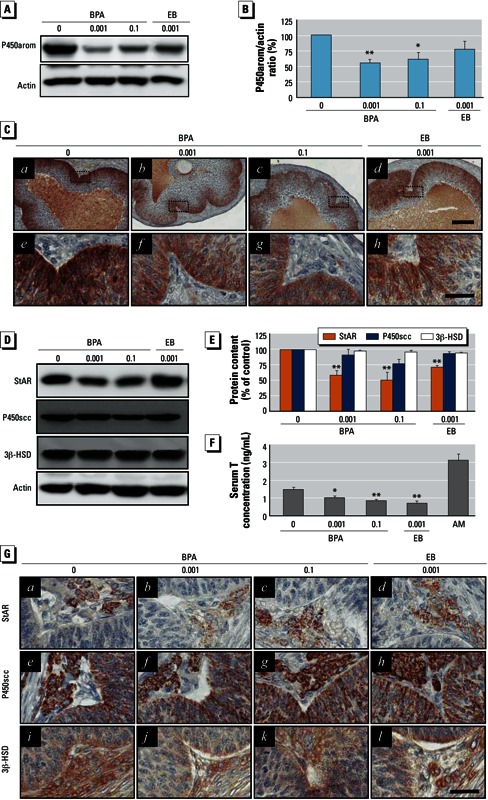
Effect of BPA exposure on P450arom in granulosa cells and StAR, P450scc, and 3β-HSD in the T-I cells of ovarian follicles. Adult female rats were adminis­tered BPA (0, 0.001, or 0.1 mg/kg BWU/day) or EB (0.001 mg/kg BW/day) for 90 days by gavage. (*A*) Western blot analysis for P450arom. (*B*) Densitometric quantification of the P450arom protein level in isolated granulosa cell protein extracts. (*C*) Immunohistochemical localization of P450arom in the granulosa cell layers of the large antral follicles; *e–h* are enlargements of the regions marked in *a–d* (original magnification: *a–d*, 100×; *e–h*, 400×; bars = 80 μm in *a–d* and 30 μm in *e–h*). (*D*) Western blot analysis for StAR, P450scc, and 3β-HSD proteins. (*E*) Densitometric quantification of StAR, P450scc, and 3β-HSD protein levels in residual ovaries. (*F*) Serum T levels (mean ± SD) measured by ELISA (*n* = 12); AM represents adult male serum (positive control). (*G*) Immunohistochemical localization of StAR, P450scc, and 3β-HSD in T-I layers (original magnification: 400×; bar = 30 μm). For *B* and *E*, data represent the mean ± SD of at least three independent experiments. *p < 0.05, and **p < 0.01 compared with control (0 mg/kg BW).

*BPA exposure and downregulation of P450arom protein expression in granulosa cells.* Ovarian aromatase expressed in granulosa cells facilitates the conversion of E2 from androgens produced in the theca cells of the antral follicles. Thus, we examined whether a change in aromatase expression was associated with E2 synthesis after BPA treatment. Decreased P450arom protein levels were evident in the granulosa cells of animals in both BPA treatment groups (Figure 3A,B). P450arom was predominantly localized in the granulosa cell layers of the large antral (preovulatory) follicles (Figure 3C). P450arom immunoreactivity was remarkably reduced in the BPA- and EB-exposed groups (Figure 3C, b–d and f–h) compared with controls (Figure 3C, a–e).

*Changes in StAR, P450scc, 3*β*-HSD, and CYP17A1 expression in T-I cells after BPA exposure*. The capability of granulosa cells to synthesize E2 is linked to the steroidogenic activity of T-I cells in terms of substrate (androgen) production and supply. Therefore, it is important to investigate the status of the major steroidogenic proteins that are involved in androgen production in these cells. We used Western blot analysis to monitor the changes in expression levels of StAR, P450scc, 3β-HSD, and CYP17A1 in residual ovaries. BPA exposure resulted in a significant downregulation of StAR expression, but P450scc and 3β-HSD levels were apparently unaffected (Figure 3D,E). CYP17A1 expression also remained unchanged after BPA exposure [see Supplemental Material, Figure S4 (http://dx.doi.org/10.1289/ehp.1205823)]. These observations were confirmed by immunohistochemistry (Figure 3G; see also Supplemental Material, Figure S4C). StAR protein was decreased in the theca cells in the BPA- and EB-exposed groups (Figure 3G, b–c). In contrast, protein immunoreactivities of P450scc (Figure 3G, f–g), 3β-HSD (Figure 3G, j and h), and CYP17A1 (see Supplemental Material, Figure S4C, b–c) were similar in the BPA- and EB-treated groupsand in the controls (Figure 3G; see also Supplemental Material, Figure S4C). Finally, we examined serum T levels in order to monitor whether substrate production of E2 is altered by decreases in StAR protein within T-I cells. We found that serum T concentration was decreased in both BPA-exposed groups (Figure 3F).

**Figure 4:**
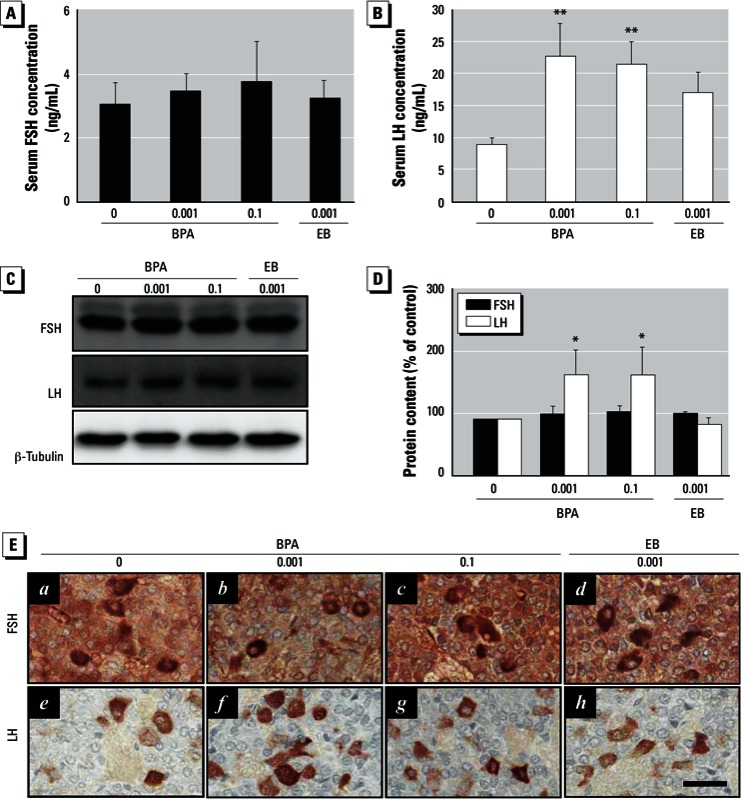
Effects of BPA exposure on serum FSH and LH levels and FSH and LH protein expression in the pituitary glands. Adult female rats were adminis­tered BPA (0, 0.001, or 0.1 mg/kg BWU/day) or EB (0.001 mg/kg BW/day) for 90 days by gavage. FSH (*A*) and LH (*B*) levels were measured by ELISA; values represent mean ± SD (*n* = 12). (*C*) Western blot analysis of FSH and LH in pituitary gland protein extracts. (*D*) Densitometric quantification of FSH and LH protein levels. Data represent the mean ± SD of at least three independent experiments. (*E*) Immunohistochemical localization of FSH and LH in the anterior pitui­tary glands (original magnification: 400×; bar = 30 μm). *p < 0.05, and **p < 0.01 compared with control (0 mg/kg BW).

*Effect of chronic BPA exposure on FSH and LH synthesis and release from the pituitary gland*. To evaluate whether the potential decrease in gonadotropin (FSH and LH) production caused by BPA exposure could result in decreased E2 synthesis in the ovary, we measured serum FSH and LH concentrations and examined FSH and LH protein expression levels in the pituitary glands. BPA exposure significantly increased serum LH levels (Figure 4B) and LH protein content in the pituitary gland (Figure 4C,D). LH immunostaining revealed that LH-positive cells were localized in the pituitary gland and that their immunoreactivities were more intense in the BPA-exposed groups (Figure 4E, f–g) than in the control group (Figure 4E, e). Serum LH levels appeared to be higher in the EB-exposed group compared with the controls (Figure 4B), but the differences were not significant. Pituitary LH protein content and immunoreactivity in the EB-exposed group did not differ from those in the controls (Figure 4C,D,E, h). In contrast to LH, neither serum FSH levels (Figure 4A), pituitary FSH protein content (Figure 4C,D), nor the immunoreactivity of FSH-positive cells in the pituitary were altered (Figure 4E, b–c).

*Short-term exposure to BPA resulted in decreased E2 via downregulation of P450arom without gonadotropin level alteration.* We performed short-term exposure experiments in adult female rats to determine whether BPA-induced E2 decreases are initially provoked by reduced pituitary gonadotropin or by follicle loss via granulosa cell apoptosis at earlier time points during BPA exposure. Both 1 and 2 weeks after BPA exposure, serum E2 concentrations were significantly decreased in the high-dose BPA- and EB-treated groups compared with controls [see Supplemental Material, Figure S5 (http://dx.doi.org/10.1289/ehp.1205823)]. Although P450arom proteins were significantly downregulated in granulosa cells after exposure to BPA, levels of StAR, P450scc, and 3β-HSD remained unchanged in residual ovaries (see Supplemental Material, Figure S6). Meanwhile, levels of LH and FSH in serum and in the pituitary were not noticeably different between controls and BPA-exposed groups (see Supplemental Material, Figure S7). During treatment, apoptotic cell death (evaluated by caspase-3 activation) was not detected in the granulosa cells of any of the groups (see Supplemental Material, Figure S6).

## Discussion

Recent studies have suggested that BPA exposure is associated with several obstetrical and gynecological problems in humans (Cantonwine et al. 2010; Kandaraki et al. 2011; Sugiura-Ogasawara et al. 2005). Given the estrogenicity of BPA, it could presumably disturb or mimic estrogen action, which is necessary for the normal maintenance of female reproduction and hormonal balance during adulthood. To date, many animal studies have focused on perinatal or neonatal BPA exposure (Adewale et al. 2009; Cabaton et al. 2011; Fernández et al. 2010; Rodríguez et al. 2010) because animals are very sensitive to exogenous chemicals during these periods. Although many women are exposed to BPA worldwide, the reproductive health risks and complications of BPA exposure during adulthood have not been evaluated. Thus, we aimed to determine whether BPA exposure during adulthood could affect ovarian steroidogenesis and subsequently provoke pathophysiological changes in the ovary.

The selection of environmentally relevant BPA doses is one of the most important factors for appropriate risk assessment of an exposure study. Generally, “low dose” is used to refer to environmentally relevant doses (i.e., doses resulting in serum levels close to those observed in human serum). In the present study, we used 1 μg/kg BW (low dose) and 100 μg/kg BW (high dose). The low dose was one-fiftieth that of the U.S. EPA reference dose (U.S. EPA 1993), and the high dose was 2 times higher than the reference dose. We believe that these doses were adequate for evaluating effects of actual environmental exposure to BPA on alterations in hormones and female reproduction *in vivo*. Furthermore, this dose range was similar to those used in previous studies (Cabaton et al. 2011; Newbold et al. 2007, 2009).

In the present study, we observed that long-term BPA exposure in adult female rats caused a significant decrease in E2 serum concentration, which was accompanied by increased duration of the estrus phase, increased ovarian cell apoptosis, and decreased E2-regulated protein expression and collagen content in the uterus. The E2 concentration decreases during the rat estrous cycle and is maintained at a relatively lower level during the estrus phase than in the other phases (i.e., diestrus and proestrus) (Kalra and Kalra 1974). Estrogen has been shown to suppress apoptosis in granulosa cells (Billig et al. 1993) and luteal cells (Goodman et al. 1998). Furthermore, E2 maintains corpus luteum function (Khan et al. 1987). Upregulation of PCNA (Rumpel et al. 1995) and calbindin-D9k (Krisinger et al. 1992) expression levels and increased collagen content (Smith and Allison, 1966) in uterine tissues are closely correlated with increased E2 levels. These findings taken together with our results indicate that serum E2 levels were indeed reduced by BPA exposure. In particular, we found that the number of caspase-3–positive apoptotic cells was significantly increased in granulosa cells of the antral follicles and in luteal cells of the corpus luteum of ovaries from rats exposed to BPA, suggesting that long-term BPA exposure during adulthood causes augmentation of follicular atresia and luteal regression in the ovary. Caspase-3 has been implicated in ovarian follicular atresia and luteal regression in the rat ovary (Boone and Tsang 1998). Although ovarian follicular atresia and luteal regression are normal physiological processes for the adequate maintenance of ovarian functions, the aberrant increase in these processes can cause disturbances in follicle selection and in the life span of corpora lutea. Several *in vitro* studies support our findings: BPA treatment resulted in decreased E2 production in FSH-stimulated porcine (Mlynarcíková et al. 2005) and human granulosa cells (Kwintkiewicz et al. 2010), and in cultured mouse antral follicles (Peretz et al. 2011). Therefore, BPA may have a direct adverse effect on the regulation of E2 production in granulosa cells.

Estrogen biosynthesis is catalyzed by P450arom (the product of the *CYP19* gene). In the ovary, P450arom expression occurs predominantly in the granulosa cells of the preovulatory follicles (Stocco 2008). Recent *in vitro* studies showed that BPA causes downregulation of *P450arom* mRNA expression in rat granulosa cells (Zhou et al. 2008), FSH-treated human (Kwintkiewicz et al. 2010) and rat (Quignot et al. 2012) granulosa cells, and mouse antral follicles (Peretz et al. 2011). Consistent with those results, we have demonstrated that long-term BPA exposure results in the downregulation of P450arom protein expression in granulosa cells of the preovulatory follicles in rats. Notably, low and high doses of BPA downregulated P450arom to the same degree, indicating that BPA doses lower than the U.S. EPA reference dose reduce E2 levels and cause subsequent changes in the female reproductive system in adult rats. Although the precise cellular and biochemical mechanism(s) underlying P450arom protein downregulation in response to BPA exposure are presently unknown, BPA presumably has a direct negative action on the transcriptional and/or translational regulation of P450arom expression in granulosa cells. Because pituitary FSH levels were unchanged in the present study, the influential effect of pituitary FSH can be excluded as a possible regulatory mechanism for P450arom expression.

Androgen production in T-I cells, which is activated by crucial steroidogenic proteins (StAR, P450acc, and 3β-HSD), is required for estrogen synthesis in the ovary (Richards 1980). In response to pituitary LH, StAR in particular transfers cholesterol from the outer membrane to the inner mitochondrial membrane (Stocco 2001), where the P450scc enzyme is located. In the present study, BPA exposure decreased StAR expression in T-I cells, but P450scc and 3β-HSD protein levels remained virtually unaffected. Previous studies have shown that BPA treatment increases *StAR* and *P450scc* mRNA expression in cultured T-I cells (Zhou et al. 2008) but decreases StAR, P450scc, and 3β-HSD in antral follicle cultures (Peretz et al. 2011). This discrepancy could originate from differences in BPA doses, treatment duration, the *in vitro* culture system, animal species, or the levels of cellular purity and differentiation. Given that serum T levels were decreased in BPA-exposed animals in the present study, we can presume that StAR is a major target protein that is affected by BPA during androgen synthesis in T-I cells.

Decreased serum levels of T and E2 ultimately stimulate the hypothalamus and pituitary gland to synthesize and release LH and FSH, respectively. Thus, the increased LH levels we observed after BPA exposure were likely associated with the decreased synthesis of T in T-I cells via homeostatic reduced negative feedback regulation. Interestingly, decreased E2 levels after BPA exposure did not induce a significant increase of FSH release from the pituitary. The interpretation of this result is difficult with the information that is currently available. However, it is tempting to speculate that the prolonged status of decreased E2 synthesis due to a lack of the substrate (T) evoked the desensitization of either hypothalamic gonadotropin-releasing hormone neurons or FSH-synthesizing gonadotrophs in the pituitary. We compared the effect of EB exposure with that of BPA throughout this study, and the results of EB treatment were similar to those in BPA-exposed groups, but were much less pronounced. In particular, EB exposure did not affect LH production in the pituitary gland. This suggests that BPA exerts a unique cytotoxic action/mechanism(s) in addition to estrogenicity. It is currently unknown whether BPA affects steroidogenesis in ovarian cells through an estrogen receptor–mediated pathway or by direct inhibitory effect on steroidogenic proteins. The possible involvement of peroxisome proliferator-activated receptor-γ (Kwintkiewicz et al. 2010) in the regulation of steroidogenesis should be further considered in regard to BPA action in the ovary.

Finally, we consistently observed a potential adverse effect of BPA on E2 production in granulosa cells, even in the short-term BPA exposure experiments, confirming the hypothesis that BPA first decreases E2 levels by directly disturbing P450arom protein expression in the granulosa cells that are dispensable for gonadotropin action. The low incidence of granulosa cell apoptosis after BPA exposure eliminates the possibility that follicle loss via caspase-3–mediated apoptosis is responsible for the reductions in E2 and the associated steroidogenic proteins. Taken together, we believe that the exposure of adult female rats to low concentrations of BPA initially reduces E2 synthesis by directly disrupting steroidogenesis within the ovary and that the prolonged status of reduced E2 levels subsequently provokes feedback regulation of LH increases as well as ovarian cell apoptosis. This new information will be a useful addition to the knowledge regarding the effects of BPA on female reproduction. Further studies on mechanism(s) by which BPA exhibits its adverse effects in ovarian cells at the molecular and biochemical levels are needed in the near future.

## Conclusion

Our data indicate that long-term exposure to environmentally relevant concentrations of BPA in adult female rats results in significant reduction of serum E2 levels and an increase in ovarian cell apoptosis, which correlates to augmentation of follicular atresia and luteal regression in the ovary. The downregulation of StAR and P450arom proteins in the ovary might be a crucial step by which E2 production is interrupted after BPA exposure. Therefore, our results suggest that BPA exposure during adulthood disturbs the maintenance of normal ovarian functions by reducing E2 and that StAR and P450arom are the definitive steroidogenic proteins that are targeted by BPA.

## Supplemental Material

(5.1 MB) PDF
