# Supplementary material for: Bisphenol A Exposure during Adulthood Causes Augmentation of Follicular Atresia and Luteal Regression by Decreasing 17β-Estradiol Synthesis via Downregulation of Aromatase in Rat Ovary
Source: Environ Health Perspect. 2013 Mar 19;121(6):663–9. doi: 10.1289/ehp.1205823 (PMC3672913; doi:10.1289/ehp.1205823)
Supplement: (5.1 MB) PDF [file ehp.1205823.s001.pdf]

Environmental Health Perspectives doi:10.1289/ehp.1205823

Supplemental Material

Bisphenol A Exposure during Adulthood Causes Augmentation of Follicular Atresia and Luteal Regression by Decreasing 17β-Estradiol Synthesis via Downregulation of Aromatase in Rat Ovary

Seung Gee Lee, Ji Young Kim, Jin-Yong Chung, Yoon-Jae Kim, Ji-Eun Park, Seunghoon Oh, Yong-Dal Yoon, Ki Soo Yoo, Young Hyun Yoo, and Jong-Min Kim

Table of contents

- Supplemental Material, Figure S1. Alterations in uterine cell proliferation and integrity following BPA exposure. --1

- Supplemental Material, Figure S2. Decreases in collagen fibers in the uterus after BPA exposure. ---2

- Supplemental Material, Figure S3. Low magnification view of immunohistochemical reactivities of active caspase-3 in the ovarian section of rats exposed to BPA. -------------3

- Supplemental Material, Figure S4. Alteration in CYP17A1 levels in the T-I cells of ovarian follicles after BPA exposure. ---4

- Supplemental Material, Figure S5. Effect of short-term BPA exposure on E2 serum concentration. ---5

- Supplemental Material, Figure S6. Changes in P450arom and active caspase-3 levels in granulosa cells and alterations in StAR, P450scc, and 3β-HSD levels in the T-I cells of ovarian follicles after short-term BPA exposure. --6

- Supplemental Material, Figure S7. Effects of short-term BPA exposure on serum FSH and LH levels and FSH and LH protein levels in the pituitary glands. ---------------------------7

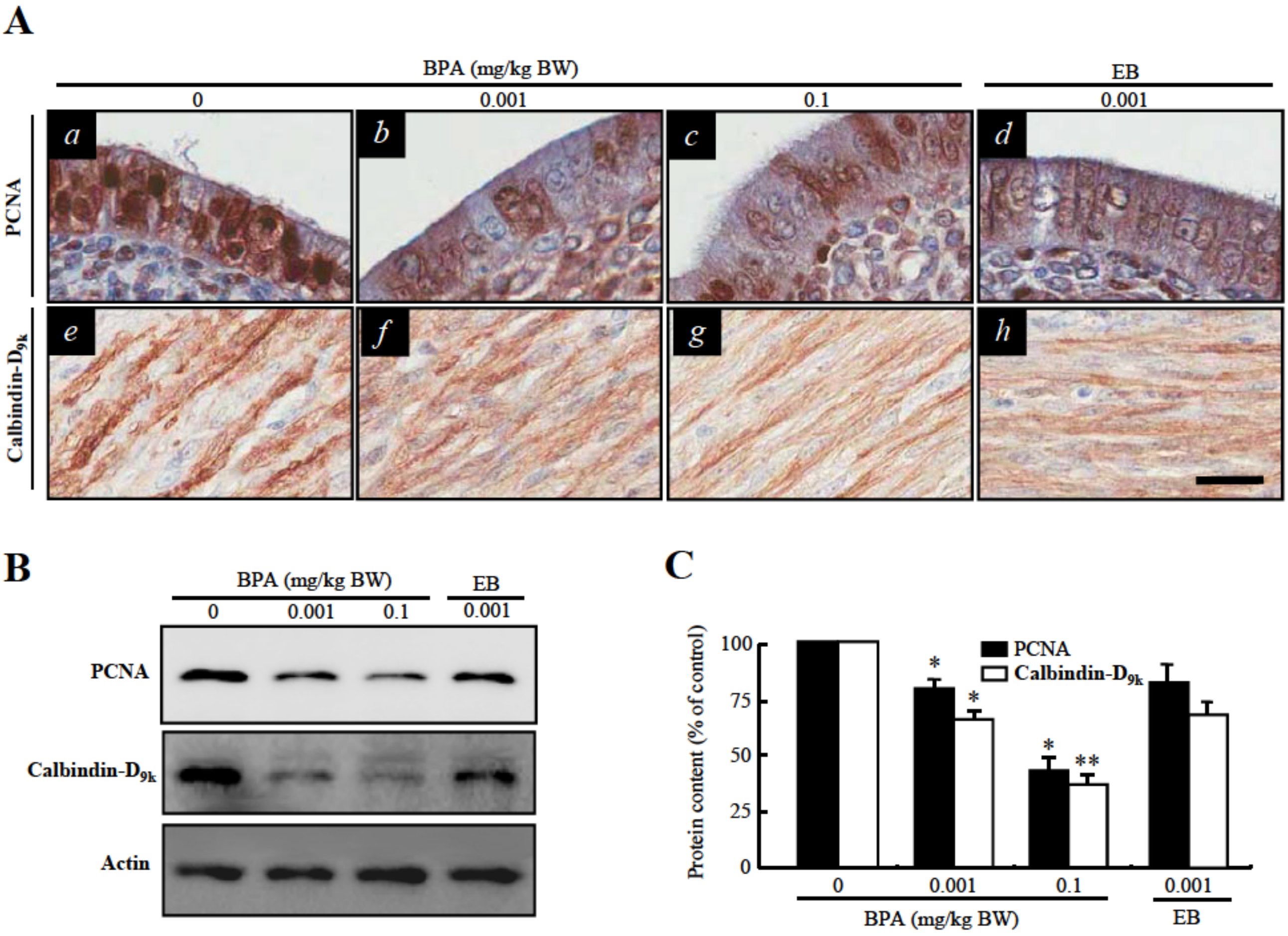

Supplemental Material, Figure S1. Alterations in uterine cell proliferation and integrity following BPA exposure. A: Immunohistochemical localizations of PCNA and calbindin-D_{9k} in uterine tissues. Original magnification: 400×; bar = 30 μm. B: Western blot analysis for PCNA and calbindin-D9k in uterine tissues. C: Densitometric quantification of PCNA and calbindin-D_{9k} protein levels in uterine tissue protein extracts. At least 3 independent experiments were performed and the data shown represents the mean ± SD. *, $P < 0.05$ and **, $P < 0.01$ compared with each control (0 mg/kg BW).

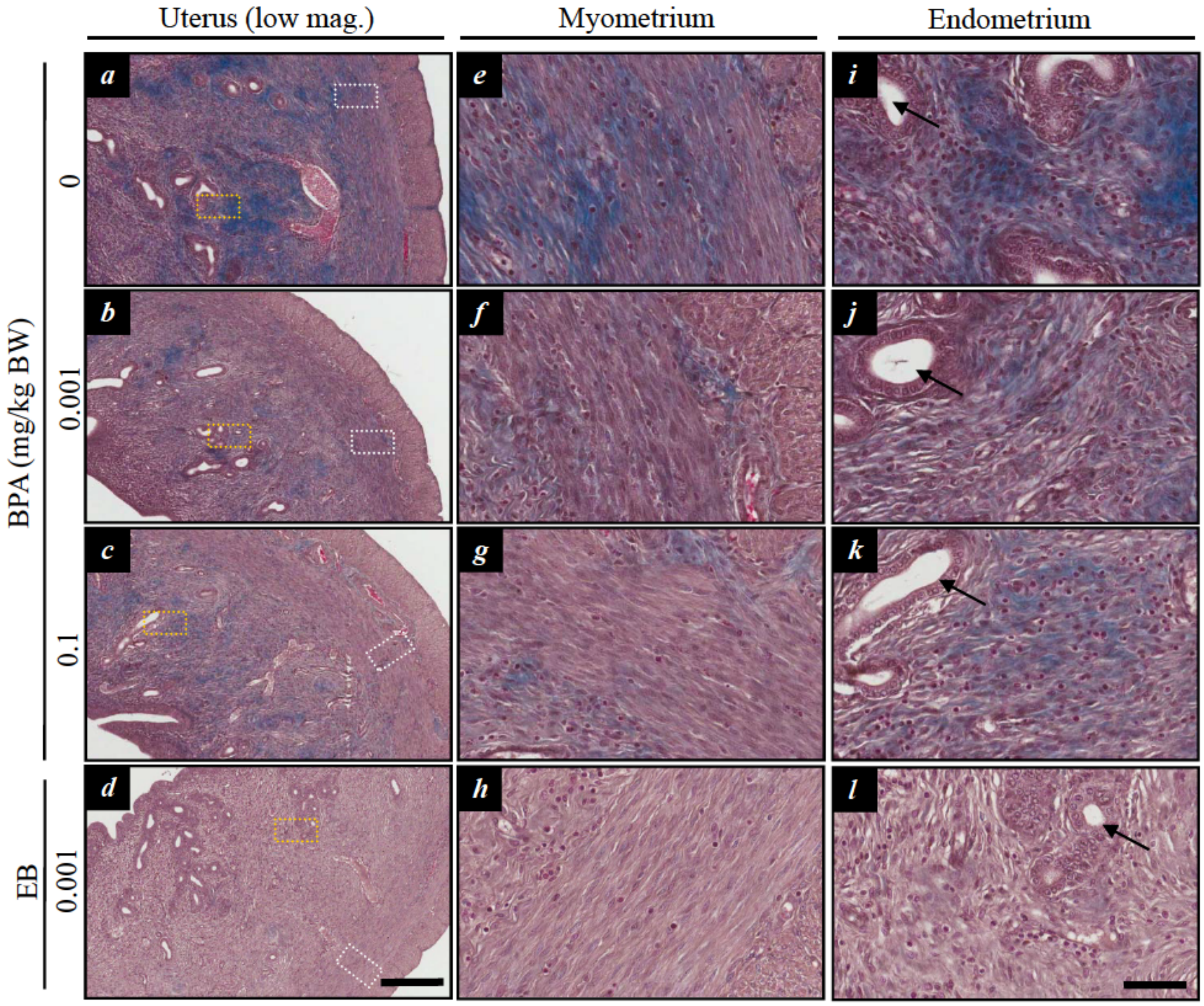

Supplemental Material, Figure S2. Decreases in collagen fibers in the uterus after BPA exposure. Left, center, and right columns represent whole uterine (*a–d*), myometrial (*e–h*), and endometrial (*i–l*) layers, respectively. Uterine sections were stained by the trichrome (Gomori) method. Collagen fibers are stained blue. Microphotographs of *e–h* an d *i–l* are enlargements of the regions marked with yellow- and white-dotted rectangular lines in a–d, respectively. Arro ws in *i–l* point to endometrial glands. Bars = 300 μm (*a-d*), 60 μm (e-l)

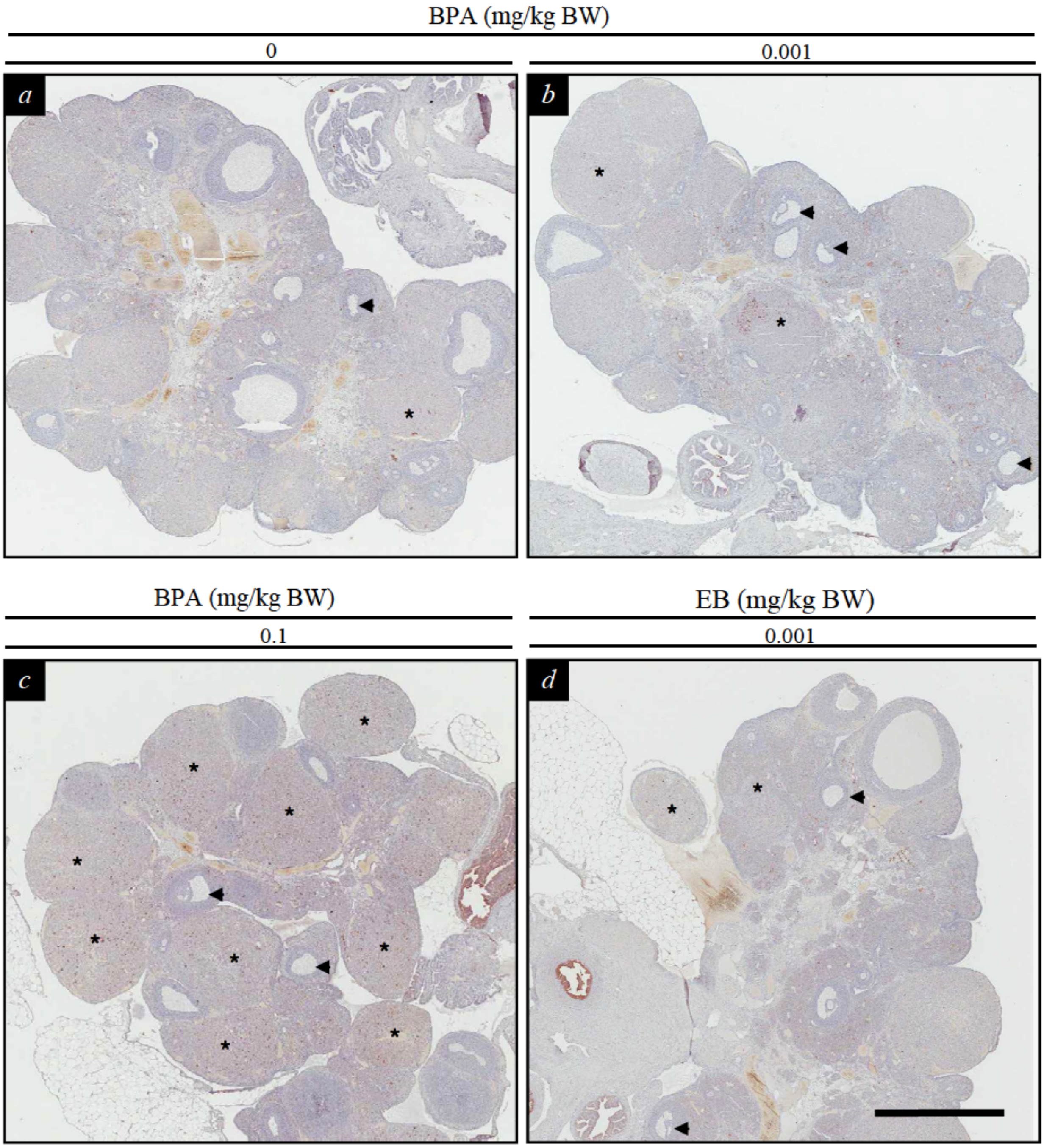

Supplemental Material, Figure S3. Low magnification view of immunohistochemical reactivities of active caspase-3 in the ovarian section of rats exposed to BPA. The rat ovary was fixed, embedded in paraffin, sectioned, and immunostained with the active form-specific antibody for caspase-3. The immunoreaction was visualized with the diaminobenzidine (DAB) reaction. Arrows point to atretic follicles that retained granulosa cells positively stained with caspase-3. Asterisks denote the regressing corpus luteum that retained luteal cells positively stained with caspase-3. Original magnification: 20×; bar = 1.2 mm.

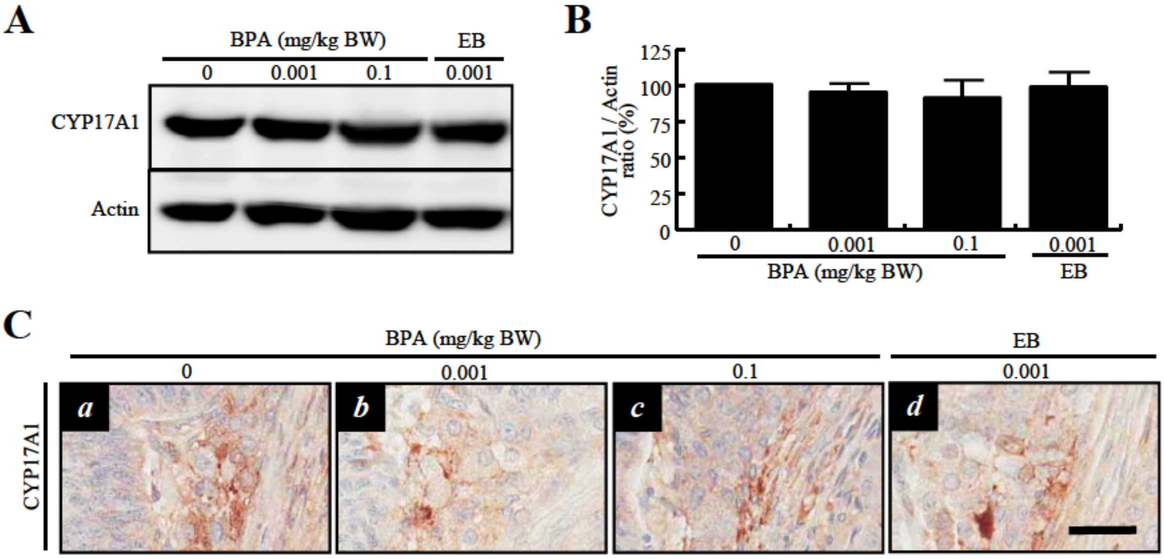

Supplemental Material, Figure S4. Alteration in CYP17A1 levels in the T-I cells of ovarian follicles after BPA exposure. A: Western blot analysis for CYP17A1 proteins. **B:** Densitometric quantification of CYP17A1 protein levels in residual ovaries. At least 3 independent experiments were performed and the data shown represent mean ± SD values. **C:** Immunohistochemical localization of CYP17A1 in T-I layers. Original magnification: 400×; bar = 30 μm.

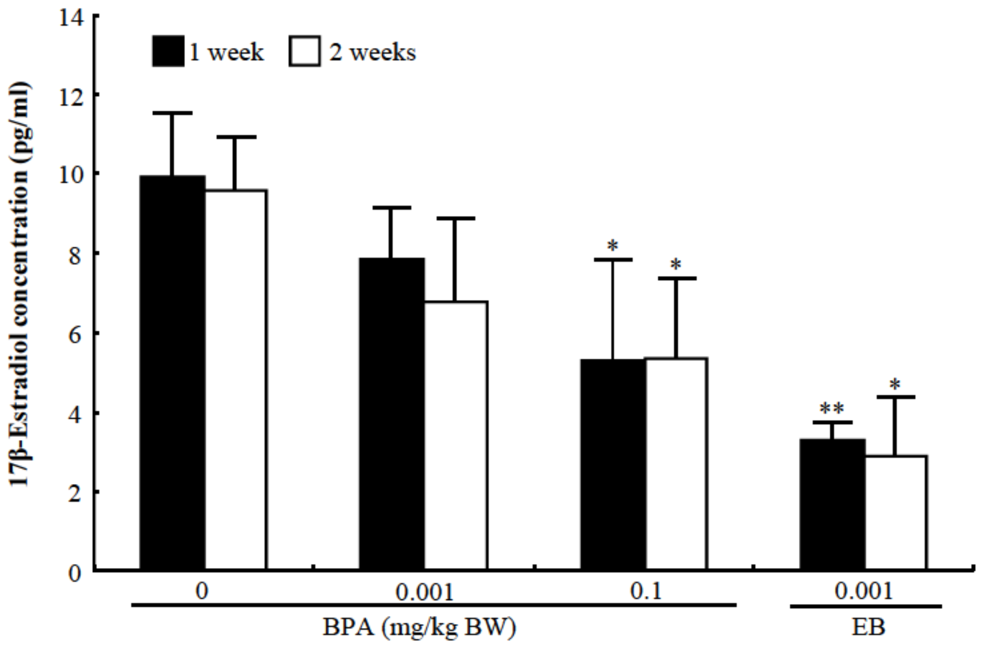

Supplemental Material, Figure S5. Effect of short-term BPA exposure on E2 serum concentration. Adult female rats received 0, 0.001, or 0.1 mg/kg BW per day of BPA or 0.001 mg/kg BW per day of EB for 1 or 2 weeks by gavage. E2 levels were measured by ELISA. Values represent the mean ± SD (n = 12). *, $P < 0.05$ and **, $P < 0.01$ compared with the control (0 m_/k_ BW).

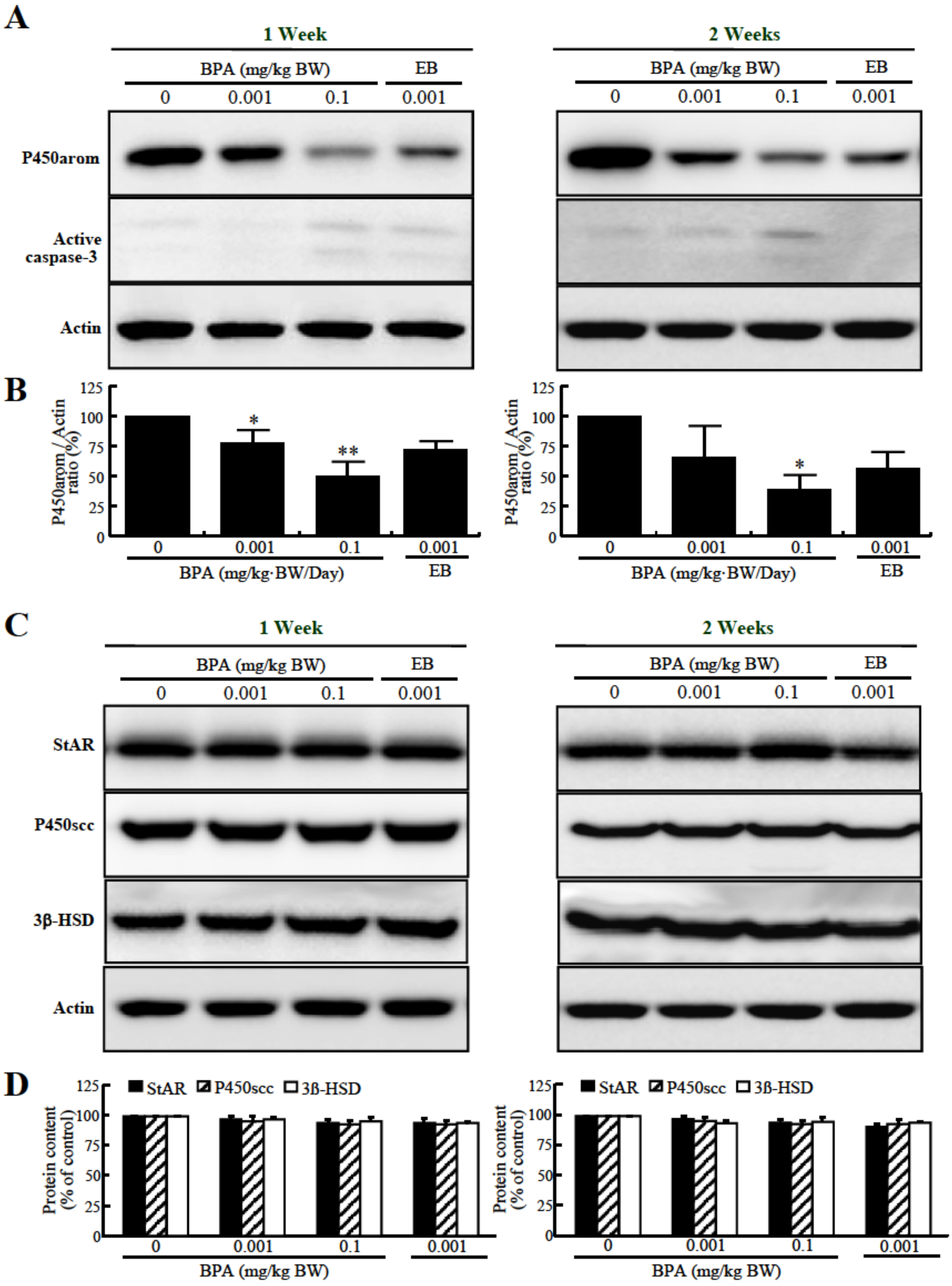

Supplemental Material, Figure S6. Changes in P450arom and active caspase-3 levels in granulosa cells and alterations in StAR, P450scc, and 3β-HSD levels in the T-I cells of ovarian follicles after short-term BPA exposure. A: Western blot analysis for P450arom and active caspase-3. **B:** Densitometric quantification of the P450arom protein levels in isolated granulosa cell protein extracts. At least 3 independent experiments were performed and the data represent mean ± SD values. *, $P < 0.05$ and **, $P < 0.01$ compared with each control (0 mg/kg BW). **C:** Western blot analysis for StAR, P450scc, and 3β-HSD proteins. **D:** Densitometric quantification of StAR, P450scc, and 3β-HSD protein levels in residual ovaries. At least 3 independent experiments were performed and the data shown represent mean ± SD values.

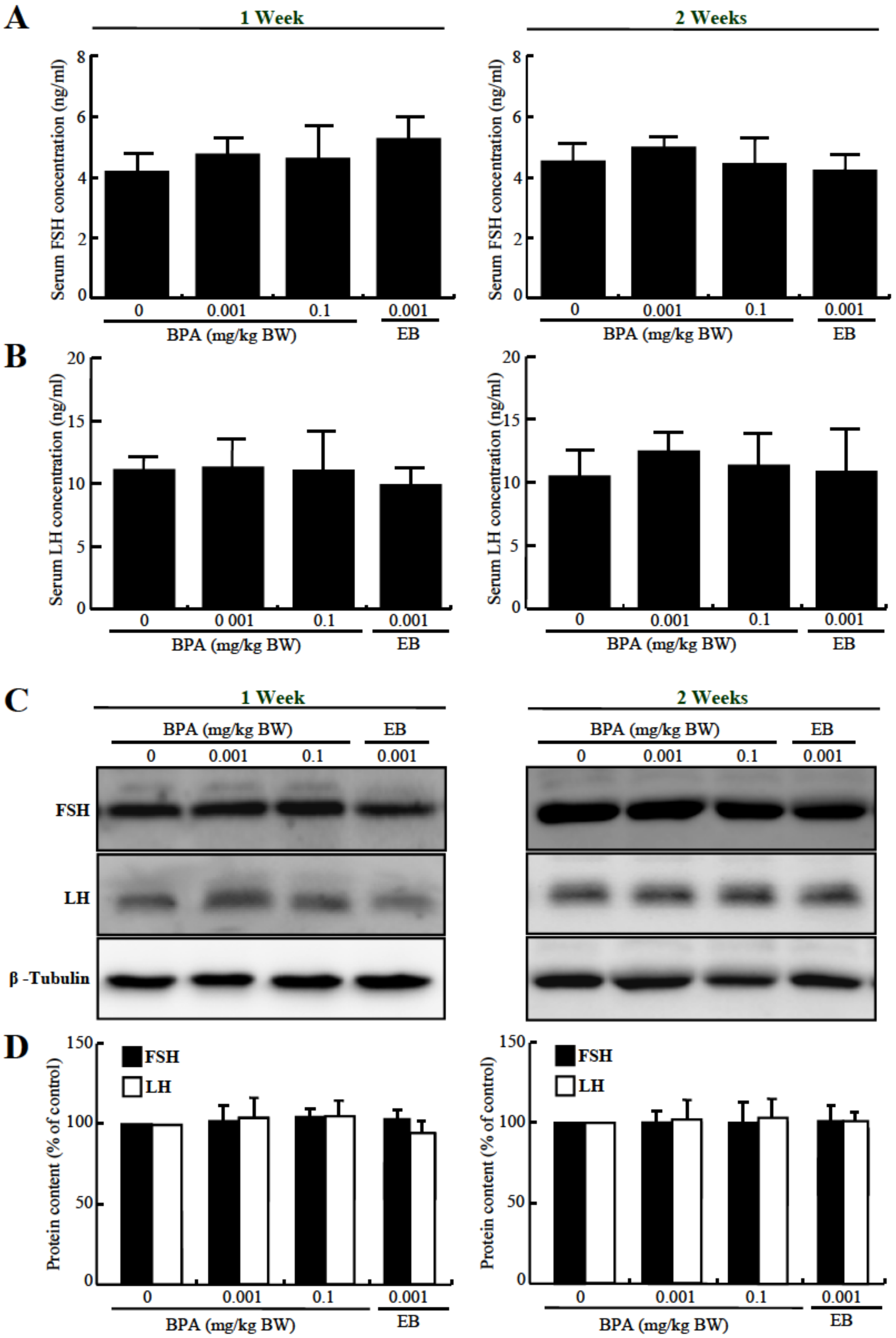

Supplemental Material, Figure S7. Effects of short-term BPA exposure on serum FSH and LH levels and FSH and LH protein levels in the pituitary glands. FSH **(A)** and LH **(B)** levels were measured by ELISA. Values represent the mean ± SD (n = 12). **C:** Western blot analysis of FSH and LH in pituitary gland protein extracts. **D:** Densitometric quantification of FSH and LH protein levels. At least 3 independent experiments were performed and the data shown represent the mean ± SD.
